# Neoadjuvant Immunotherapy Combined Chemotherapy Followed by Surgery Versus Surgery Alone for Locally Advanced Esophageal Squamous Cell Carcinoma: A Propensity Score-Matched Study

**DOI:** 10.3389/fonc.2021.797426

**Published:** 2021-12-14

**Authors:** Zhi-Nuan Hong, Kai Weng, Kaiming Peng, Zhen Chen, Jihong Lin, Mingqiang Kang

**Affiliations:** ^1^ Department of Thoracic Surgery, Fujian Medical University Union Hospital, Fuzhou, China; ^2^ Key Laboratory of Cardio-Thoracic Surgery, Fujian Medical University, Fujian Province University, Fuzhou, China; ^3^ Key Laboratory of Ministry of Education for Gastrointestinal Cancer, Fujian Medical University, Fuzhou, China; ^4^ Fujian Key Laboratory of Tumor Microbiology, Fujian Medical University, Fuzhou, China

**Keywords:** esophagectomy, neoadjuvant immunotherapy, surgery, esophageal cancer squamous cell carcinoma, operation difficulty

## Abstract

**Background:**

Combination of neoadjuvant immunotherapy and chemotherapy (nICT) is a novel treatment for locally esophageal cancer squamous cell carcinoma (ESCC). This study aimed to evaluate the potential effect of nICT on surgery safety by comparing short-term outcomes between the surgery alone group and the nICT followed by surgery group.

**Methods:**

A retrospective analysis was performed to identify patients (from January 2017 to July 2021) who underwent surgery for ESCC with or without nICT. A propensity score matching (PSM) comparison (1:1) was conducted to reduce selection biases and balance the demographic and oncologic characteristics between groups.

**Results:**

After PSM, the nICT group (*n* = 38) was comparable to the surgery alone group (*n* = 38) in the following characteristics: age, sex, BMI, ASA status, smoking, tumor location, lymph node resection, clinical stage, anastomotic location, surgical approach, and surgical approach. The operation time and incidence of postoperative pneumonia in the nICT group were higher than those in the control group (*p* < 0.05). However, other complications and major complications were comparable between the two groups. There was no significant difference between the two groups in intraoperative blood loss, ICU stay time, postoperative hospital stay, and hospitalization cost. The 30-day mortality, 30-day readmission, and ICU readmission rates were also similar in the nICT and control groups. In the nICT group, the pathological complete response rate in primary tumor was 18.4%, and the major pathological response rate in tumor was 42.1%.

**Conclusions:**

Based on our preliminary experience, nICT followed by surgery is safe and effective with acceptable increased operation risk, manageable postoperative complications, and promising pathological response. Further multicenter prospective trials are needed to validate our results.

## Background

Esophageal cancer (EC) is one of the most prevalent types of cancer and a major cause of death with 572,000 new diagnosis cases and 500,000 deaths annually. Esophageal cancer squamous cell carcinoma (ESCC) is the main sub-type in Asians (1, 2). Esophagectomy plays an important role in treatment for locally advanced ESCC (3). However, surgery alone is often associated with high recurrence and metastasis rates up to 43.3%–50.0% (4).

Compared to surgery alone, neoadjuvant chemotherapy (nCT) and neoadjuvant chemoradiotherapy (nCRT) have been proven to improve long-term survival without additional postoperative morbidity and mortality (5). In Asia, nCT followed by surgery has been advocated as standard treatment for locally advanced ESCC (6). Kamarajah et al. reported that compared to nCT, overall survival benefit was evident for nCRT (HR 0.78, 0.62 to 0.97), and recommended nCRT followed by surgery for ESCC (6). A meta-analysis including 4,529 patients (nCT: 2,035; nCRT: 2,494) found that compared to the nCT group, deaths caused by tumor progression or recurrence were significantly less in the nCRT group than in the nCT group; however, there was not an increase in 5-year survival (7). Optimal neoadjuvant treatment strategy for locally advanced ESCC is still controversial and not promising. It is necessary to explore novel treatment regiments to achieve better long-term prognosis (8).

Antibodies against the immune inhibitory pathway of programmed death 1 (PD-1) protein or PD-1 ligand 1 (PD-L1) checkpoint inhibitors is a milestone in treatment of ESCC. In CheckMate 577 trails, Kelly et al. reported that nivolumab adjuvant therapy could prolong 11.4 months disease-free survival among patients with resected esophageal or gastroesophageal junction cancer who had received nCRT (9). Recently, pembrolizumab plus chemotherapy has been recommended as first-line treatment for advanced EC (10). Considering the promising results in advanced EC, it is reasonable to explore the efficacy and safety of neoadjuvant immunotherapy combined chemotherapy (nICT) (11, 12).

Recently, Shen et al. reported a cohort of 27 patients who received surgery after 2 cycles of nICT with a low-toxicity profile, a high R0 resection rate, and a promising pathological complete response (pCR) rate (13). Although the nICT has become popular, there were still concerns that immune therapy may affect surgical safety, complications, and mortality. To date, there was only a handful of study focusing on the above concerns and there remains a need for further evidence. This study aimed to evaluate the potential effect of nICT on surgery by comparing short-term outcomes in the surgery alone group and esophagectomy followed by nICT.

## Methods

### Patient Selection and Study Design

This study was approved by the Ethics Committee of Fujian Medical University Union Hospital. Patients’ written informed consent was obtained. Consecutive patients were recruited retrospectively who underwent esophagectomy with or without nICT for ESCC at Fujian Medical University Union Hospital from January 2017 to July 2021. The inclusion criteria were as follows: (1) thoracic ESCC; (2) receiving minimally invasive esophagectomy (MIE); and (3) with complete clinical data. Patients with nonresectable tumors or metastases during exploratory surgery or who received either neoadjuvant chemotherapy or chemoradiotherapy were excluded.

A propensity score matched analysis (1:1) was conducted to balance the demographic and oncologic characteristics. Propensity score was measured based on 4 factors: age, BMI, clinical tumor-lymph node-metastasis (cTNM) stage (for nICT group: cTNM stage after neoadjuvant therapy), and American Society of Anesthesiologists (ASA) status. We chose cTNM stage after neoadjuvant therapy due to two reasons: First, before treatment, most patients in the nICT group were diagnosed with III or IV cTNM stage, and it is hard to conduct a balanced match with the surgery alone group. Second, compared with using cTNM stage before neoadjuvant therapy, using cTNM stage after neoadjuvant therapy could better reflect the clinical reality, and further confirm the safety and efficacy of nICT followed by surgery. The 8th edition American Joint Committee on Cancer/Union for International Cancer Control staging system was used in clinicopathologic staging.

### Treatment Protocols

Patients who meet the following inclusions received nICT: (1) aged between 18 and 75 years old; (2) staged as cT_1-2_N_1-3_M_0_ or cT_3-4a_N_0-3_M_0_; (3) with normal hematologic, hepatic, and renal function; and (4) ECOG status ranged 0–2. The patients received 2–4 cycles of intravenous PD-1 inhibitor (sintilimab at a dose of 200 mg, pembrolizumab at a dose of 200 mg, and camrelizumab at a dose of 200 mg) every 3 weeks (day 1). Chemotherapy mainly consisted of simultaneous treatment with platinum-based drugs and paclitaxel [TP regimen, with cisplatin (60 mg/m^2^) on day 1, and albumin-bound paclitaxel (125 mg/m^2^) on days 1 and 8]. Surgery was performed within 4–8 weeks after the end of the last neoadjuvant treatment. All patients received MIE with standard 2-field or 3-field lymphadenectomy and gastric reconstruction. We regularly conducted standard 2-field lymphadenectomy. Neck lymphadenectomy was conducted when patients were suspected with swollen lymph nodes in the neck.

### Outcome Measures

Postoperative complications in hospital were coded using the Clavien-Dindo classification; major complications were defined as Clavien-Dindo classification grade ≧ 3 (14). The primary end point was 30-day complications. Secondary end points were interval to surgery, operation time, thoracic drainage tube stay, 30-day readmission rate, and 30-day mortality. Interval to surgery was defined as the last measured from the end of last neoadjuvant treatment to the date of surgery. Operative time was measured from incision to wound closure. ICU stay was defined as from the day of entry into the ICU to the day of leaving the ICU.

### Statistical Analysis

Patients were classified into two groups, the surgery alone group and the nICT group. The propensity score (PS) matched analysis was used to reduce the bias. PS was calculated with a logic model to fit the following variables: age, sex, BMI, and tumor-node-metastasis (TNM) stage. Setting caliper = 0.05, matching ratio = 1:1, and two comparable groups of patients were created with 38 patients in each group. The continuous variable of normal distribution was expressed as mean ± standard deviation, the continuous variable of abnormal distribution was expressed as median (quartile range), and the classified variable was expressed as number (percentage). For equivalent variables with a normal distribution, an independent Student’s *t*-test was used. The Mann–Whitney *U* test was used to compare the abnormal distribution variables between the two groups. The frequency of the classification variables was determined by using Pearson 2 or Fisher’s exact test, where appropriate. Statistical analysis was conducted in R version 4.0.4 (R Foundation for Statistical Computing, Vienna, Austria). A two-sided *p*-value < 0.05 was considered as significant.

## Results

### Patient Selection and Baseline Characteristics

To reduce the confounding bias, we conducted a 1:1 PSM cohort between the nICT group (*n* = 38) and surgery alone group (*n* = 38). After PSM, the clinical and demographic characteristics of the two groups were well balanced, including age, gender, BMI, ASA status, hypertension history, smoking, tumor location, lymphadenectomy, pathological stage, anastomotic position, route of gastric conduit, procedure type, and operative approach. The baseline characteristics are summarized in **Table 1**.

**Table 1:** Baseline characteristics after propensity score matching.

Characteristics	nICT group	Control group	*p*
Number	38	38	NA
Age	58.8 ± 7.6	59.1 ± 7.91	0.9
Male	22	21	0.74
BMI	22.4 ± 1.9	22.9 ± 3.1	0.57
ASA			0.60
1	1	0	
2	35	36	
3	2	2	
Diabetes	2	1	0.60
Hypertension	4	6	0.50
Smoking history	24	22	0.64
FEV1	2.8 ± 0.6	2.5 ± 0.6	0.28
EF%	67.8 ± 6.2	67.7 ± 5.9	0.94
Neoadjuvant cycle	2 (2, 2)	NA	NA
Interval to surgery	46.5 ± 19.1	NA	NA
Tumor location			0.17
Upper	1	3	
Middle	21	26	
Lower	16	9	
cTNM Stage (nICT Group: stage after neoadjuvant therapy)			0.21
I	19	16	
II	4	10	
III	15	12	
IV	0	0	
Lymphadenectoy			0.08
2-field	35	38	
3-field	3	0	
Anastomotic position			NA
Cervical	38	38	
Thoracic	0	0	
Route of gastric conduit			0.08
Posterior mediastinal	38	35	
Restro-sternal	0	3	
Procedure type			0.64
Robot-assisted	35	36	
Thoracoscopy	3	2	

ASA, American Society of Anesthesiologists; BMI, body mass index; FEV1, Forced expiratory volume in one second; EF, Ejection Fractions. NA, Not available.

### Complications and Short-Term Outcomes

All patients successfully received MIE. No patients converted to open surgery. The nICT group had a significantly longer operation time (311.7 ± 74.5 min), compared to that in the surgery alone group (273.4 ± 51.5 min). The number of removed lymph nodes were more in the nICT group, with a median 35.5 and 30 in the nICT group and surgery alone group, respectively (*p* = 0.039). The intraoperative blood loss was comparable. The nICT group had more thoracic drainage volume (*p* = 0.25). Furthermore, the thoracic drainage tube stay was significantly longer in the nICT group (*p* < 0.001). The ICU stay, hospital stay, postoperative hospital stays, hospital cost, 30-day mortality, 30-day readmission, and ICU readmission were similar in both groups. Perioperative outcomes before and after PSM are summarized in **Table 2**.

**Table 2:** Perioperative outcomes after propensity score matching.

Outcomes	nICT group	Control group	*p*
Operative time (min)	311.7 ± 74.5	273.4 ± 51.5	0.01
Converted to open surgery	0	0	NA
Intraoperative blood loss (ml)	100 (50, 100)	100 (80, 100)	0.77
Lymph nodes moved number	35.5 (28.3, 42)	30(21.8, 37.8)	0.039
Thoracic drainage tube stay (days)	7 (8, 12.5)	4 (3, 5.3)	<0.001
Thoracic drainage volume (ml)	1,895 (1,150, 2,675)	1,500 (1,173.5, 1,086.5)	0.25
ICU stay (days)	0 (0, 0.5)	0 (0, 0)	0.38
ICU readmission (*n*)	2	1	0.39
30-day mortality (*n*)	0	0	NA
30-day readmission (*n*)	2	2	1.00
Postoperative hospital stay (days)	9 (9, 14.3)	10 (8, 14.5)	1.00
Hospital stay (days)	21.5 (15.8, 28.5)	19 (16, 25)	0.49
Hospital cost (10,000 RMB)	8.8 (7.9, 11.3)	8.9 (8.4, 10.9)	0.82

Hb, hemoglobin; ICU, intensive care unit; CCI, comprehensive complications index. NA, Not available.

**Figure 1:**
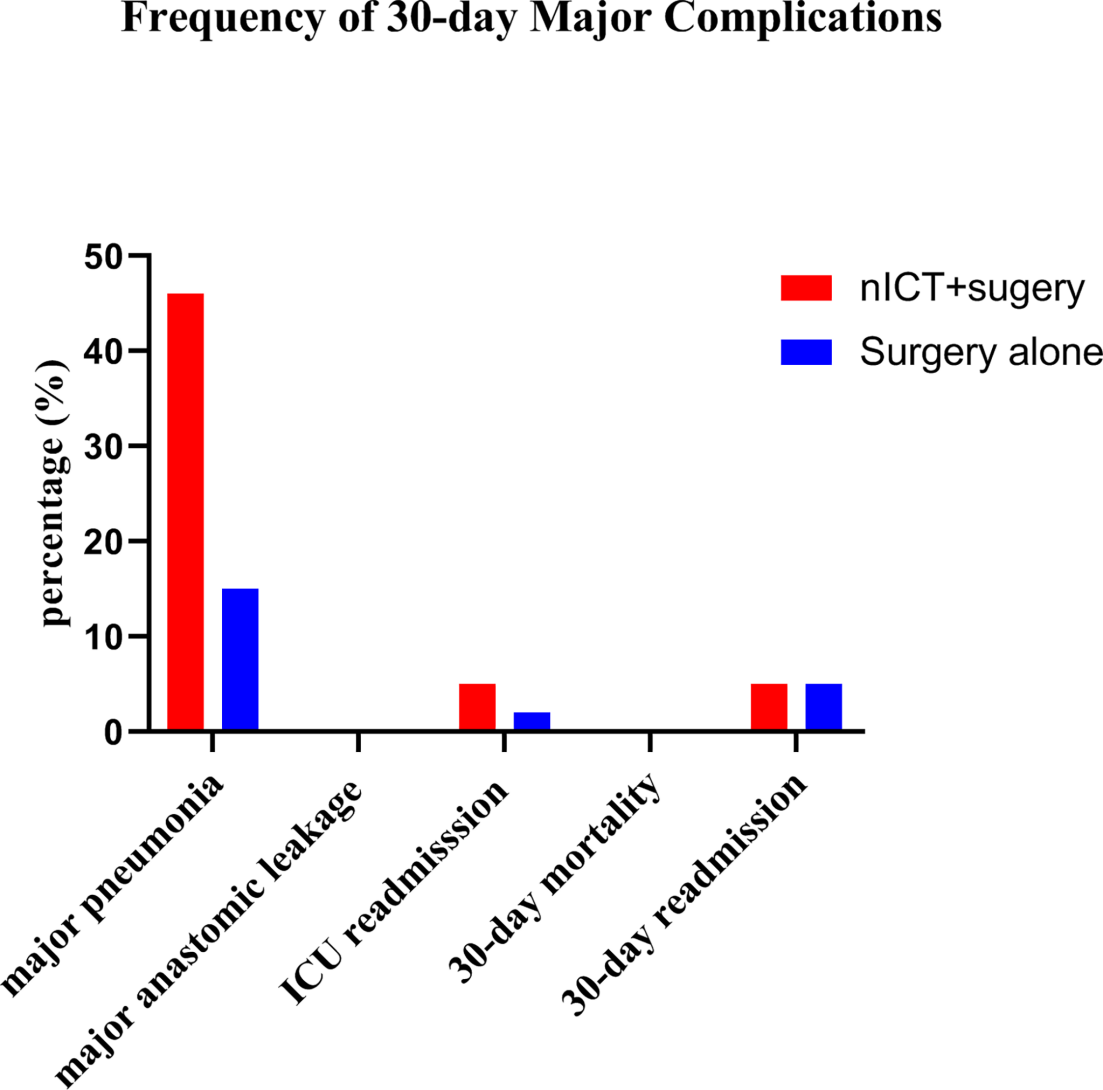
Frequencies of 30-day major complications (defined as Clavien-Dindo classification grade ≧ 3) after propensity score matching in the neoadjuvant immunotherapy combined chemotherapy (nICT) group and the surgery alone group.

Complications within 30 days after PSM are summarized in **Table 3**. Incidences of anastomotic leakage, pleural effusion, palsy of recurrent laryngeal nerve, chylothorax, bleeding, and postoperative blood transfusion were similar in nICT group and surgery alone group. The incidence of pneumonia was significantly higher in the nICT group (24/38, 63.2%) than that in the surgery alone group (11/38, 28.9%). Frequency of 30-day major complications after PSM is listed in **Figure 1**.

**Table 3:** Postoperative complications within 30-day after operation coded by the Clavien-Dindo classification.

Complications	nICT group	Control group	*p*
Pneumonia			0.005
Grade 0	14	27	
Grade 2	6	5	
Grade 3	11	6	
Grade 4	7	0	
Anastomotic leakage			0.18
Grade 0	35	31	
Grade 1	0	0	
Grade 2	3	7	
Grade 3	0	0	
Pleural effusion			0.16
Grade 0	19	23	
Grade 1	13	9	
Grade 2	3	0	
Grade 3	3	6	
Palsy of recurrent laryngeal nerve			1.00
Grade 0	36	36	
Grade 1	2	2	
Cardiac events			0.06
Grade 0	29	36	
Grade 1	2	0	
Grade 2	7	2	
Chylothorax			0.16
Grade 0	36	37	
Grade 1	2	1	
Bleeding			0.33
Grade 0	37	38	
Grade 1	0	0	
Grade 2	1	0	
Postoperative blood transfusion			1.00
Grade 0	37	37	
Grade 2	1	1	

### Efficacy

**Figure 2:**
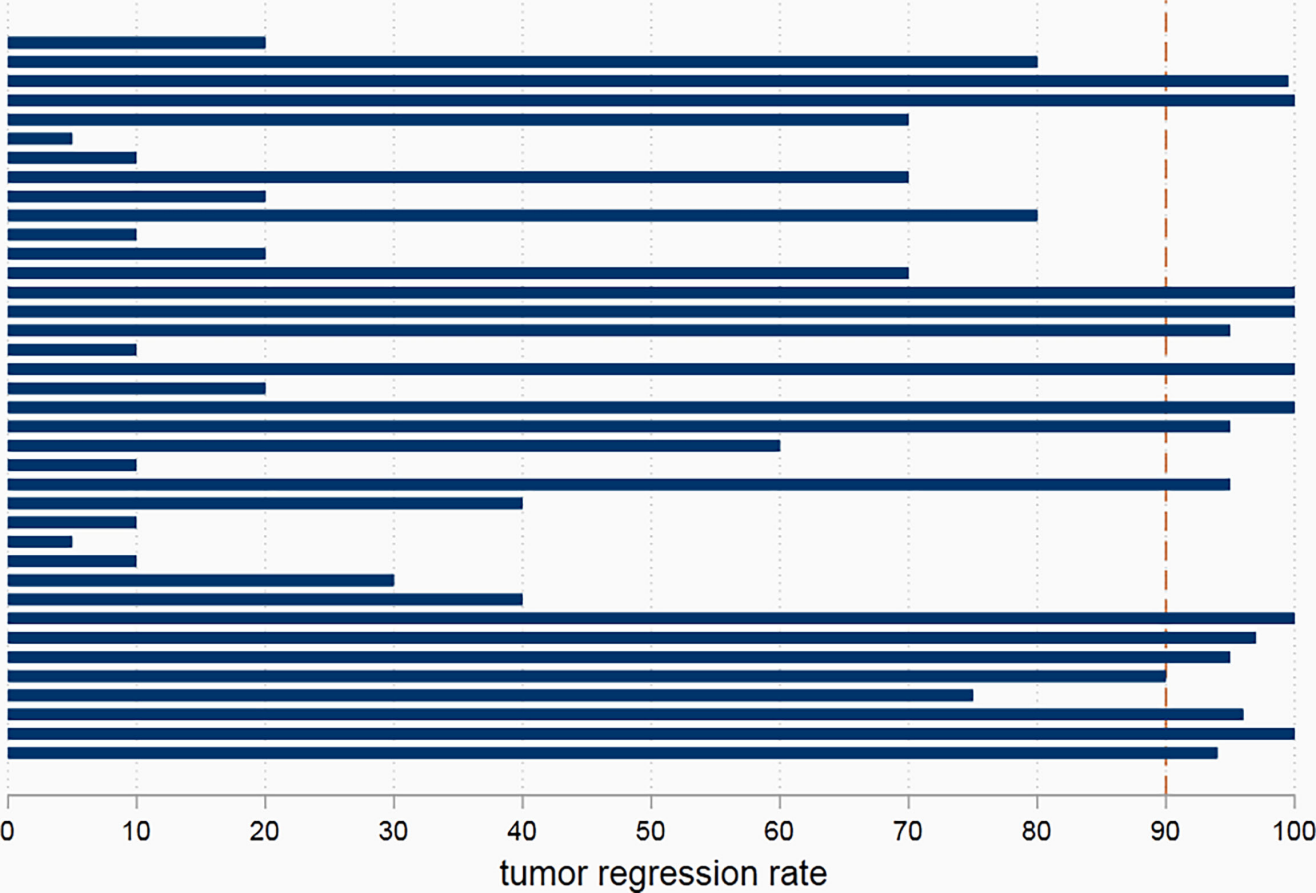
The pathological tumor regression rate in patients receiving neoadjuvant immunotherapy combined chemotherapy.

R0 resection was achieved in all patients in the nICT group and control group. In the nICT cohort, 21 patients achieved clinical partial recovery, and 17 patients achieved clinical stable disease. In the surgery alone group, 1 patient achieved clinical partial advance; others achieved clinical stable disease during the period of waiting for surgery. In the nICT group, seven patients (7/38, 18.4%) achieved pCR in primary tumors, and sixteen patients (16/38, 42.1%) achieved major pathological response (MPR) in primary tumors. Two patients still had cancer residual in lymph node, while achieving pCR in primary tumors. The median tumor regression rate was 72.5%. The details of tumor regression are shown in **Figure 2**.

## Discussion

In this study, there were no significant postponement of surgery after completion of neoadjuvant therapy. Although the operation time in the nICT group was longer than that in control, which contributed to a higher incidence of pneumonia in nICT group, other major complications were comparable between the nICT group and control group after PSM. Furthermore, there was no significantly increased risk of 30-day mortality, 30-day readmission, and ICU readmission due to neoadjuvant immunotherapy. The postoperative hospital stay and hospital cost were similar in the nICT group and control group. From our preliminary experience, nICT followed by surgery is safe and effective with acceptable increased operation risk and manageable postoperative complications.

Operation time, especially duration of one-lung ventilation (OLV), is a risk factor for postoperative pneumonia. OLV is necessary to help achieve optimal surgical exposure during thoracic surgery and reduce the contralateral lung contamination (15). However, OLV can cause serious physiological disorders. Then, the ventilated lung is exposed to hyperperfusion and ventilator-induced lung injury, whereas the collapsed lung is mainly affected by ischemia-reperfusion injury (16). Lai et al. found that OLV ≥150 min is an important risk factor for postoperative pneumonia after McKeown esophagectomy and recommended that lung protection should be taken when OLV prolongation is expected (17). We found that incidence of major pneumonia was similar in the nICT group and control group. The possible reason is that early intervention could treat pneumonia. In our institution, we positively managed preoperative comorbidities, such as diabetes and chronic obstructive pulmonary disease (18), and advise patients to do respiratory function exercise. Sputum suction was conducted during operation and after operation. Early diagnosis of pneumonia and antibiotic treatment can prevent progression to respiratory failure and an increased risk of death. For patients who received nICT, pneumonitis may occur after operation, and sometimes it is difficult to diagnose based on CT scan. One patient underwent pneumonitis on the 7th day after operation, and this patient finally recovered with methylprednisolone treatment. Thus, for patients who have received nICT, pneumonitis should be considered when postoperative pneumonia does not respond to antibiotic treatment. Totally, the postoperative complications in nICT group were manageable.

In this study, we attributed the higher incidence of postoperative pneumonia to the longer operation time in the nICT group. From our experience, the thoracic surgery led to a longer operation time. Previous studies on the safety and feasibility of surgical resection after neoadjuvant immunotherapy for non-small cell lung cancer have shown increased surgical difficulty and technical challenges. Chaft et al. reported that response to immunotherapy in patients with non-small cell lung cancer may lead to high-density fibrosis. For patients with dense fibrosis (19), dissection of the mediastinum and hilum is technically challenging. Bott et al. reported the possibility of an unexpected transition from thoracoscopic lobectomy after immunotherapy (20), although there was still no report of an unexpected transformation from thoracoscopic surgery to open surgery in patients receiving nICT. However, the adhesions at the site of tumor retreat would result in unclear interstitial boundaries, especially if the tumor is located in the middle thoracic region adjacent to the trachea. Thus, when there is an adhesion between the esophagus and surrounding tissue, the surgeon needs to carefully distinguish the tissue boundaries to avoid damage to trachea, thoracic duct, and important nerve and vessels. For surgeons still on a learning curve, esophagectomy on patients who received nICT is not recommended. Fortunately, there were no dense adhesions that would require unexpected thoracotomy. Sihag et al. conducted a PSM to compare the short-term outcomes between the neoadjuvant immunotherapy and chemoradiotherapy, and the chemoradiotherapy alone group. Based on their preliminary experience, esophagectomy is safe and feasible following combined neoadjuvant immunotherapy and standard chemoradiotherapy for locally advanced esophageal cancer (21). Thus, from our opinions, the operation risk and technical challenge from immune therapy were acceptable.

Hao Wang et al. reported the pCR rate of resected tumors of 35.7% in the nCRT group and 3.8% in the nCT group (22). In this study, the anti-tumor effect of nICT were promising, with a pCR rate of 18.4% in primary tumor and a MPR rate in tumor 42.1%. Pathological response to neoadjuvant therapy is significantly associated with long-term survival in patients with ESCC (23). It seems that a combination of immune therapy appears to be superior to that with nCT only and inferior to that in nCRT. Considering that most patients in this study were at the clinical III or IVA stage, there is a need to conduct long-term follow-up to evaluate the efficacy of nICT pattern.

The current study is known to have limitations. Its retrospective design introduces the inevitable risk of selection and information bias. Although propensity score matching was used to minimize indication confusion, potential bias can never be completely eliminated. All operations were performed by specialized, experienced thoracic surgeons in high-volume centers with surgery volume over 300 cases per year to ensure the treatment standardization. The sample size is relatively limited, and a multi-center large-sample study is needed to further verify the current results.

## Conclusion

Compared with the control group, esophagectomy followed by nICT would increase operation time and incidence of pneumonia. However, the nICT group and control group were similar in major postoperative complications and mortality. Based on our preliminary experience, nICT followed by surgery is safe and effective with acceptable increased operation risk and promising pathological response.

## Publisher’s Note

All claims expressed in this article are solely those of the authors and do not necessarily represent those of their affiliated organizations, or those of the publisher, the editors, and the reviewers. Any product that may be evaluated in this article, or claim that may be made by its manufacturer, is not guaranteed or endorsed by the publisher.

## Data Availability Statement

The raw data supporting the conclusions of this article will be made available by the authors, without undue reservation.

## Author Contributions

Z-NH and MK conceived the concept and coordinated the design. Z-NH drafted the manuscript with significant contributions from KP and KW. All authors contributed to the article and approved the submitted version.

## Funding

This study was sponsored by Key Laboratory of Cardio-Thoracic Surgery (Fujian Medical University), Fujian Province University.

## Conflict of Interest

The authors declare that the research was conducted in the absence of any commercial or financial relationships that could be construed as a potential conflict of interest.

## Publisher’s Note

All claims expressed in this article are solely those of the authors and do not necessarily represent those of their affiliated organizations, or those of the publisher the editors and the reviewers. Any product that may be evaluated in this article, or claim that may be made by its manufacturer, is not guaranteed or endorsed by the publisher.
